# Utility and clinical effectiveness of liquid biopsy-based DNA methylation testing in early tumor screening and diagnosis, emphasizing older adult populations: a systematic review and meta-analysis

**DOI:** 10.3389/fonc.2025.1739963

**Published:** 2026-01-12

**Authors:** Yujuan Gao, Haoyu Xu, Juan Li, Minli Li, Desheng Zhu

**Affiliations:** 1 Department of Radiation Oncology, Tai’an Cancer Hospital, Tai’an, China; 2 Department of Gastroenterology and Hepatology, Nanjing Jinling Hospital, Affiliated Hospital of Medical School, Nanjing University, Nanjing, China; 3 Department of Radiation Oncology, Jinling Hospital, Affiliated Hospital of Medical School, Nanjing University, Nanjing, China; 4 Department of Urology, Affiliated Jinhua Hospital, Zhejiang University School of Medicine, Jinhua, Zhejiang, China

**Keywords:** aging population, CDO1, DNA methylation, early diagnosis, liquid biopsy, lung cancer

## Abstract

**Background:**

The global aging population faces a disproportionate burden of lung cancer, creating an urgent need for minimally invasive screening methods. Liquid biopsy-based DNA methylation testing, particularly targeting the cysteine dioxygenase type 1 (CDO1) gene promoter, has emerged as a promising approach for early cancer detection. This study aims to systematically evaluate the diagnostic value of CDO1 promoter methylation in lung cancer using liquid biopsies.

**Methods:**

A comprehensive literature search was conducted across PubMed/MEDLINE, Embase, Web of Science, and Cochrane Library from inception to 28Th September, 2025. Studies reporting CDO1 promoter methylation in liquid biopsies (blood, urine, or sputum) for lung cancer detection with histological confirmation were included. Methodological quality was assessed using QUADAS-2. Bivariate random-effects models were used to calculate pooled sensitivity, specificity, diagnostic odds ratio (DOR), positive/negative likelihood ratios (PLR/NLR), and 95% confidence intervals.

**Results:**

Seven studies involving 655 lung cancer cases and 402 controls were included. CDO1 methylation demonstrated pooled sensitivity of 0.72 and specificity of 0.89 for blood-based liquid biopsies, and 0.66 and 0.87 for non-blood specimens, respectively. The overall diagnostic odds ratio was 19.13 (95% CI: 13.53-26.84), with positive and negative likelihood ratios of 7.73 (95% CI: 5.77-10.35) and 0.32 (95% CI: 0.26-0.39). No significant heterogeneity was observed across studies (I² = 0% for all parameters). Subgroup analyses revealed consistent performance across different specimen types and geographic regions.

**Conclusion:**

CDO1 promoter methylation detection in liquid biopsies shows robust diagnostic accuracy for lung cancer, with performance characteristics supporting its potential clinical utility, particularly for older populations who face barriers to conventional screening. The consistency across diverse specimen types and populations underscores its promise as a minimally invasive screening tool.

## 1 Introduction

The global population is undergoing a significant demographic transition toward older age, a shift that carries profound implications for public health, particularly in the field of oncology (1). Age stands as the single most dominant risk factor for cancer development. This strong correlation is rooted in biological realities: the cumulative lifetime exposure to carcinogens, the gradual decline in immune system efficacy known as immunosenescence, and the accumulated somatic alterations in cells over time. As a direct consequence, older adults face a disproportionately high and growing burden of cancer, often presenting with advanced disease and experiencing poorer outcomes. This pressing reality demands a critical re-evaluation of our current approaches to cancer control, with a specific focus on developing early detection strategies that are both effective and suitable for an aging demographic (2).

The established paradigm for cancer screening relies on modalities such as low-dose computed tomography for lung cancer, mammography for breast cancer, and colonoscopy for colorectal cancer (3). While these methods have demonstrated success in reducing mortality, their limitations become particularly pronounced when applied to older populations. Imaging techniques can generate a high number of false positive results, leading to unnecessary invasive procedures, patient anxiety, and increased healthcare costs. Endoscopic procedures, though effective, are inherently invasive and carry risks of complications that can be more severe for patients with comorbidities. Furthermore, this model of modality specific screening creates a fragmented system where an individual must undergo multiple separate tests, a process that is logistically challenging, physically demanding, and often results in lower adherence among older adults.

The clear shortcomings of these conventional methods have catalyzed the search for a better alternative: a minimally invasive, broadly applicable, and highly accurate biomarker. The field of liquid biopsy, which analyzes tumor-derived materials in blood, initially focused on detecting genetic mutations in circulating tumor DNA. However, this approach faces a significant challenge in the context of aging. A common age-related condition called clonal hematopoiesis can lead to non-cancerous blood cells acquiring mutations that mimic cancer signals, resulting in false positive results. This biological confounder is especially prevalent in the elderly population, precisely the group targeted for screening, thereby limiting the reliability of mutation-based liquid biopsies.

In light of these challenges, scientific attention has turned decisively toward DNA methylation, a fundamental epigenetic mechanism. DNA methylation involves the addition of a methyl group to cytosine bases in specific genomic regions and is crucial for regulating gene expression (4). In cancer, this precise epigenetic landscape becomes profoundly distorted. Tumors often exhibit hypermethylation, which silences critical tumor suppressor genes, alongside global hypomethylation that promotes genomic instability. For diagnostic purposes, methylation patterns offer a distinct advantage over mutations (5). While mutations are scattered randomly across the genome, aberrant methylation frequently occurs in specific, recurrent regions called CpG islands. This predictable nature makes methylation an ideal and stable target for clinical assay development. Moreover, methylation patterns are highly tissue specific, providing a molecular signature that can not only detect cancer but also predict its tissue of origin, a critical feature for guiding subsequent clinical workup (6).

The convergence of the biological strengths of DNA methylation with the practical benefits of liquid biopsy creates a powerful synergy. Tumors continuously shed DNA fragments into the bloodstream, and a fraction of this cell-free DNA originates from the tumor itself, carrying its unique methylation signature (7). Modern technologies can now detect these minute, cancer-specific epigenetic signals against the background of normal DNA. This advancement has unlocked the potential for multi-cancer early detection tests. Such tests aim to screen for multiple cancer types from a single, simple blood draw (8). For an older individual, this represents a potential paradigm shift. It could replace a daunting array of separate tests with a single, low-risk procedure that can be easily integrated into a routine clinical visit, thereby overcoming major barriers to screening adherence (9).

Despite this considerable promise, the translation of methylation based liquid biopsy into routine clinical practice, especially for early cancer detection in older adults, remains a work in progress (10). Its true clinical utility and effectiveness must be rigorously demonstrated. Critical questions persist. How sensitive and specific are these tests for detecting early stage, localized disease in older populations? How does their performance vary across different cancer types and across different age strata within the elderly? (11) The higher prevalence of cancer in older adults increases the positive predictive value of a test, which is beneficial. However, this same characteristic demands an exceptionally high specificity to prevent an overwhelming number of false positives, which could lead to unnecessary, invasive, and potentially harmful diagnostic procedures in a vulnerable population. Therefore, a comprehensive evaluation must extend beyond analytical performance to assess the impact on patient outcomes, integration into existing care pathways, and the psychological and economic consequences of implementation (12).

In summary, the challenge posed by demographic aging calls for innovative, pragmatic, and patient-centric solutions in early cancer detection. The limitations of current screening methods are acutely felt by older adults. Liquid biopsy based DNA methylation testing emerges as a transformative technology that leverages the recurrent and tissue specific nature of epigenetic alterations (13). It holds the unique potential to enable efficient, multi-cancer screening that is particularly suited to the physiological and logistical realities of the elderly. Realizing this full potential, however, hinges on rigorous clinical validation and health services research that explicitly addresses its utility, effectiveness, and cost-effectiveness within this specific, high-risk demographic (14). This review aims to synthesize the current evidence and explore the trajectory of this rapidly evolving field, with a focused emphasis on its profound implications for transforming cancer care and public health strategy for our aging populations (15).

## 2 Methods

### 2.1 Search strategy and data sources

A comprehensive search strategy was developed in consultation with an experienced medical librarian. Systematic searches were conducted across multiple electronic databases including PubMed/MEDLINE, Embase, Web of Science, and Cochrane Central Register of Controlled Trials from their inception through 28^Th^ September, 2025. To minimize publication bias and identify ongoing or unpublished studies, we also searched clinical trial registries (ClinicalTrials.gov, WHO International Clinical Trials Registry Platform) and preprint servers (medRxiv, bioRxiv). The search strategy combined controlled vocabulary terms and keywords related to “lung neoplasms”, “CDO1 protein”, “DNA methylation”, and “liquid biopsy” using appropriate Boolean operators. No language restrictions were applied. Additional studies were identified by scanning reference lists of included articles and relevant systematic reviews.

Search Strategy in pubmed as following:

#1 “Lung Neoplasms”[mh] OR “Carcinoma, Non-Small-Cell Lung”[mh] OR “Carcinoma, Small Cell Lung”[mh] OR “Pulmonary Neoplasms”[tiab] OR “Lung Cancer”[tiab] OR “Lung Carcinoma”[tiab] OR NSCLC[tiab] OR SCLC[tiab]

#2 “CDO1 protein, human”[Supplementary Concept] OR “CDO1”[tiab] OR “Cysteine Dioxygenase Type 1”[tiab] OR “Cysteine Dioxygenase”[tiab]

#3 “DNA Methylation”[mh] OR “CpG Islands”[mh] OR “Promoter Regions, Genetic”[mh] OR “DNA Methylation”[tiab] OR “Methylation”[tiab] OR “Promoter Methylation”[tiab] OR “Epigenetic”[tiab] OR “Hypermethylation”[tiab]

#4 “Liquid Biopsy”[mh] OR “Circulating Tumor DNA”[mh] OR “Cell-Free Nucleic Acids”[mh] OR “Liquid Biopsy”[tiab] OR “Liquid Biopsies”[tiab] OR “ctDNA”[tiab] OR “circulating tumor DNA”[tiab] OR “cell-free DNA”[tiab] OR “cfDNA”[tiab] OR “Blood-Based”[tiab] OR “Plasma”[tiab] OR “Serum”[tiab]

#5 #1 AND #2 AND #3 AND #4

Embase:

#1 ‘lung tumor’/exp OR ‘non small cell lung cancer’/exp OR ‘small cell lung cancer’/exp OR ‘pulmonary neoplasms’:ti,ab OR ‘lung cancer’:ti,ab OR ‘lung carcinoma’:ti,ab OR ‘nsclc’:ti,ab OR ‘sclc’:ti,ab

#2 ‘cdo1 protein’/exp OR ‘cdo1’:ti,ab OR ‘cysteine dioxygenase type 1’:ti,ab OR ‘cysteine dioxygenase’:ti,ab

#3 ‘dna methylation’/exp OR ‘cpg island’/exp OR ‘promoter region’/exp OR ‘dna methylation’:ti,ab OR ‘methylation’:ti,ab OR ‘promoter methylation’:ti,ab OR ‘epigenetic’:ti,ab OR ‘hypermethylation’:ti,ab

#4 ‘liquid biopsy’/exp OR ‘circulating tumor dna’/exp OR ‘cell free dna’/exp OR ‘liquid biopsy’:ti,ab OR ‘liquid biopsies’:ti,ab OR ‘ctdna’:ti,ab OR ‘circulating tumor dna’:ti,ab OR ‘cell-free dna’:ti,ab OR ‘cfdna’:ti,ab OR ‘blood-based’:ti,ab OR ‘plasma’:ti,ab OR ‘serum’:ti,ab

#5 #1 AND #2 AND #3 AND #4

Web of Science:

#1 TS=(“lung neoplasms” OR “pulmonary neoplasms” OR “lung cancer” OR “lung carcinoma” OR “NSCLC” OR “SCLC”)

#2 TS=(“CDO1” OR “Cysteine Dioxygenase Type 1” OR “Cysteine Dioxygenase”)

#3 TS=(“DNA Methylation” OR “Methylation” OR “Promoter Methylation” OR “Epigenetic” OR “Hypermethylation”)

#4 TS=(“Liquid Biopsy” OR “Liquid Biopsies” OR “ctDNA” OR “circulating tumor DNA” OR “cell-free DNA” OR “cfDNA” OR “Blood-Based” OR “Plasma” OR “Serum”)

#5 #1 AND #2 AND #3 AND #4

### 2.2 Study selection criteria

Studies were selected based on predetermined PICOS criteria. The population comprised patients with suspected or confirmed lung cancer. The index test was CDO1 promoter methylation analysis in liquid biopsy specimens. The reference standard was histological confirmation of lung cancer. Eligible studies needed to provide sufficient data to construct 2×2 contingency tables for diagnostic accuracy calculations. We included both prospective and retrospective observational studies with case-control or cohort designs. No studies were excluded based solely on their prospective or retrospective nature to ensure a comprehensive evidence synthesis. However, we acknowledge that case-control designs, which were included, are inherently susceptible to spectrum and selection biases compared to cohort studies. Exclusion criteria included case reports, reviews, conference abstracts, and studies with overlapping populations or insufficient data.

### 2.3 Data extraction and management

Two independent investigators extracted data using a piloted data extraction form. The extracted information included: first author, publication year, study design, patient characteristics, sample size, specimen type, CDO1 methylation detection method, threshold values, and diagnostic accuracy data. For studies that reported results for multiple methylation thresholds, we prioritized data from the threshold that was pre-specified by the authors as primary or, if none was specified, the threshold that provided the best balance between sensitivity and specificity as determined by the Youden index. If studies reported results from multiple specimen types from the same patient cohort, we included only one specimen type per study in the primary meta-analysis to avoid double-counting of patients. We prioritized plasma over serum and blood-based specimens over non-blood specimens to maintain consistency across studies, as plasma was the most commonly reported biospecimen. Corresponding authors were contacted via email for missing or unclear data. Of the seven studies included, three required additional data clarification. We received responses from two authors, providing the requested information. For the one non-responsive case, we utilized the available published data and conducted a sensitivity analysis excluding this study to assess its impact on the pooled estimates. The results remained consistent, confirming the robustness of our findings. Any discrepancies in data extraction were resolved through consensus or arbitration by a third investigator.

### 2.4 Quality assessment

Methodological quality was assessed independently by two reviewers using the Quality Assessment of Diagnostic Accuracy Studies 2 (QUADAS-2) tool. This instrument evaluates four domains: patient selection, index test, reference standard, and flow and timing. Each domain was rated for risk of bias and concerns regarding applicability using signaling questions. Disagreements were resolved through discussion or by consulting a third reviewer.

### 2.5 Statistical analysis and data synthesis

We employed a bivariate random-effects model to calculate pooled estimates of sensitivity, specificity, positive and negative likelihood ratios, and diagnostic odds ratios with corresponding 95% confidence intervals. This model accounts for the inherent, often negative, correlation between sensitivity and specificity across studies by incorporating a random effect for each study and modeling the two outcomes (logit-transformed sensitivity and logit-transformed specificity) jointly. We assumed an unstructured variance-covariance matrix for the random effects, allowing the estimation of between-study heterogeneity (τ²) for each outcome and their correlation (ρ). To explicitly address the potential influence of study design on diagnostic accuracy estimates, we conducted a pre-specified subgroup analysis comparing prospective versus retrospective studies. This allowed us to empirically assess whether the design contributed to heterogeneity or bias in the pooled results. Furthermore, the risk of bias within individual studies was rigorously assessed using the QUADAS-2 tool, and the overall impact of methodological quality on the meta-analysis conclusions was considered during interpretation.

### 2.6 Assessment of heterogeneity and subgroup analysis

Heterogeneity was comprehensively evaluated using I² statistics and Cochran’s Q test, with I² values of 25%, 50%, and 75% representing low, moderate, and high heterogeneity, respectively. To investigate potential sources of heterogeneity, we conducted pre-specified subgroup analyses stratified by: (1) study design (prospective vs. retrospective); (2) specimen type (plasma vs. serum); (3) methylation detection method (qMSP vs. ddPCR vs. NGS-based approaches); (4) cancer stage (early-stage I-II vs. advanced-stage III-IV); (5) age distribution (studies with mean age ≥ 70 years vs. < 70 years); (6) geographic region (Asian vs. non-Asian populations); and (7) methylation threshold (high vs. low stringency). The significance of subgroup differences was tested using meta-regression models.

### 2.7 Sensitivity analysis and publication bias

Sensitivity analyses were performed by sequentially excluding each study to assess the stability of the pooled results. Additionally, we conducted sensitivity analyses restricted to studies with low risk of bias. Publication bias was assessed using Deeks’ funnel plot asymmetry test, with a p-value < 0.10 indicating significant asymmetry. All analyses were performed using Stata version 17.0 with the “midas” package.

## 3 Result

### 3.1 Study selection

**Figure 1:**
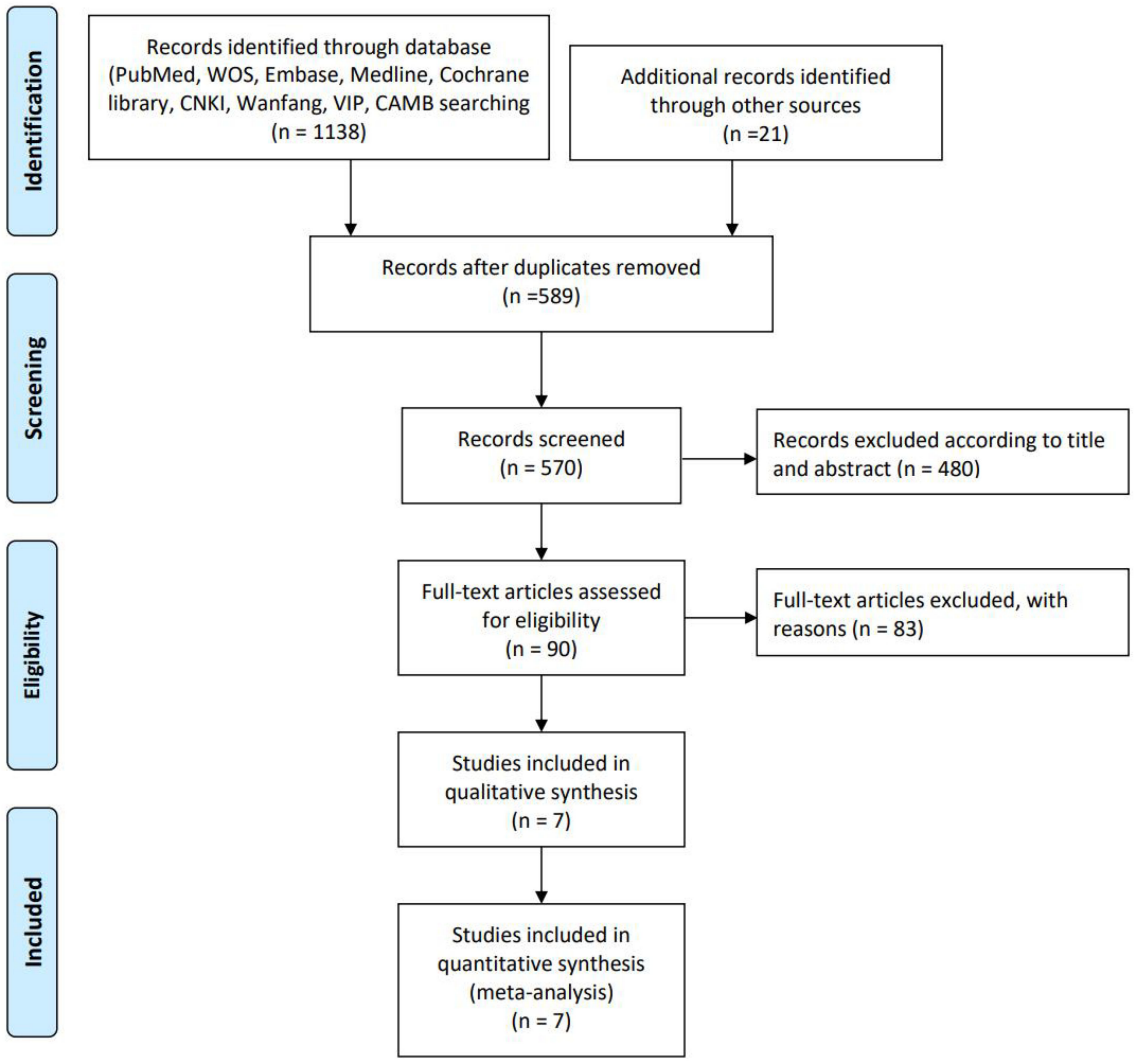
PRISMA flow diagram of the study selection process.

The systematic literature search identified a total of one thousand one hundred fifty-nine records from various sources. Electronic databases yielded one thousand one hundred thirty-eight citations, while additional sources contributed twenty-one records. After removing five hundred sixty-nine duplicate entries, five hundred eighty-nine unique records advanced to the screening phase. During the title and abstract screening phase, four hundred eighty records were excluded for not meeting the inclusion criteria. The remaining ninety articles proceeded to full-text review for eligibility assessment. Through this detailed evaluation, eighty-three studies were excluded with specific reasons, resulting in seven studies that fully satisfied all inclusion criteria. These seven studies were included in both the qualitative synthesis and quantitative meta-analysis. The complete study selection process is detailed in the PRISMA flow diagram (**Figure 1**).

### 3.2 Characteristic of the included studies

**Table 1** summarizes the characteristics of four studies utilizing blood-based liquid biopsies for CDO1 methylation analysis in lung cancer detection. These studies were conducted across three countries (China, USA, and Korea) between 2017 and 2024, encompassing a total sample size of 505 cases and 305 controls. The studies investigated lung cancer patients across various TNM stages (I-IV), with particular representation of lung adenocarcinoma (LUAD) and lung squamous cell carcinoma (LUSC). All four studies employed plasma as the biospecimen and utilized either quantitative methylation-specific PCR (qMSP) or quantitative PCR (qPCR) as detection methods (**Table 1**).

**Table 1:** Characteristics of studies using blood-based liquid biopsies (plasma).

Author (year)	Country	TNM stage	LUSC	LUAD	SCLC	Others	Sample size (case/control)	Detection method
Wang et al., 2020 (16)	China	I-IV	29	112	14	23	178/173	qPCR
Liu et al., 2020 (17)	USA	I-IV	9	65	/	/	74/25	qMSP
Chen et al., 2017 (18)	China	I	35	/	/	/	163/83	qMSP
Park et al., 2024 (19)	Korea	I-IV	42	53	26	4	90/24	qMSP

**Table 2** presents three studies employing non-blood liquid biopsies from urine and sputum samples. These studies, conducted from 2017 to 2022, included 150 cases and 97 controls from the Netherlands, China, and USA. The research focused primarily on early-stage lung cancer (stages I-III), with one study specifically investigating stage I non-small cell lung cancer patients with very small nodules. The detection methodologies consistently used methylation-specific PCR analysis across all three studies, demonstrating the feasibility of CDO1 methylation detection in alternative liquid biopsy sources beyond blood (**Table 2**).

**Table 2:** Characteristics of studies using non-blood liquid biopsies.

Author (year)	Country	TNM stage	LUSC	LUAD	SCLC	Others	Sample source	Sample size (case/control)
Wever et al., 2022 (20)	The Netherlands	I-III	16	27	/	1	Urine	44/50
Chen et al., 2020 (21)	China	I	22	139	/	2	Urine	71/27
Hulbert et al., 2017 (22)	USA	I-II	26	121	/	3	Sputum	35/20

Regarding the timing of liquid biopsy collection, five studies utilized samples collected at the time of or prior to initial diagnosis, while two studies used pre-treatment samples from confirmed lung cancer patients. No studies included samples collected during or after systemic therapy, minimizing the potential confounding effect of treatment on methylation levels.

### 3.3 Risk of bias assessment

**Figure 2:**
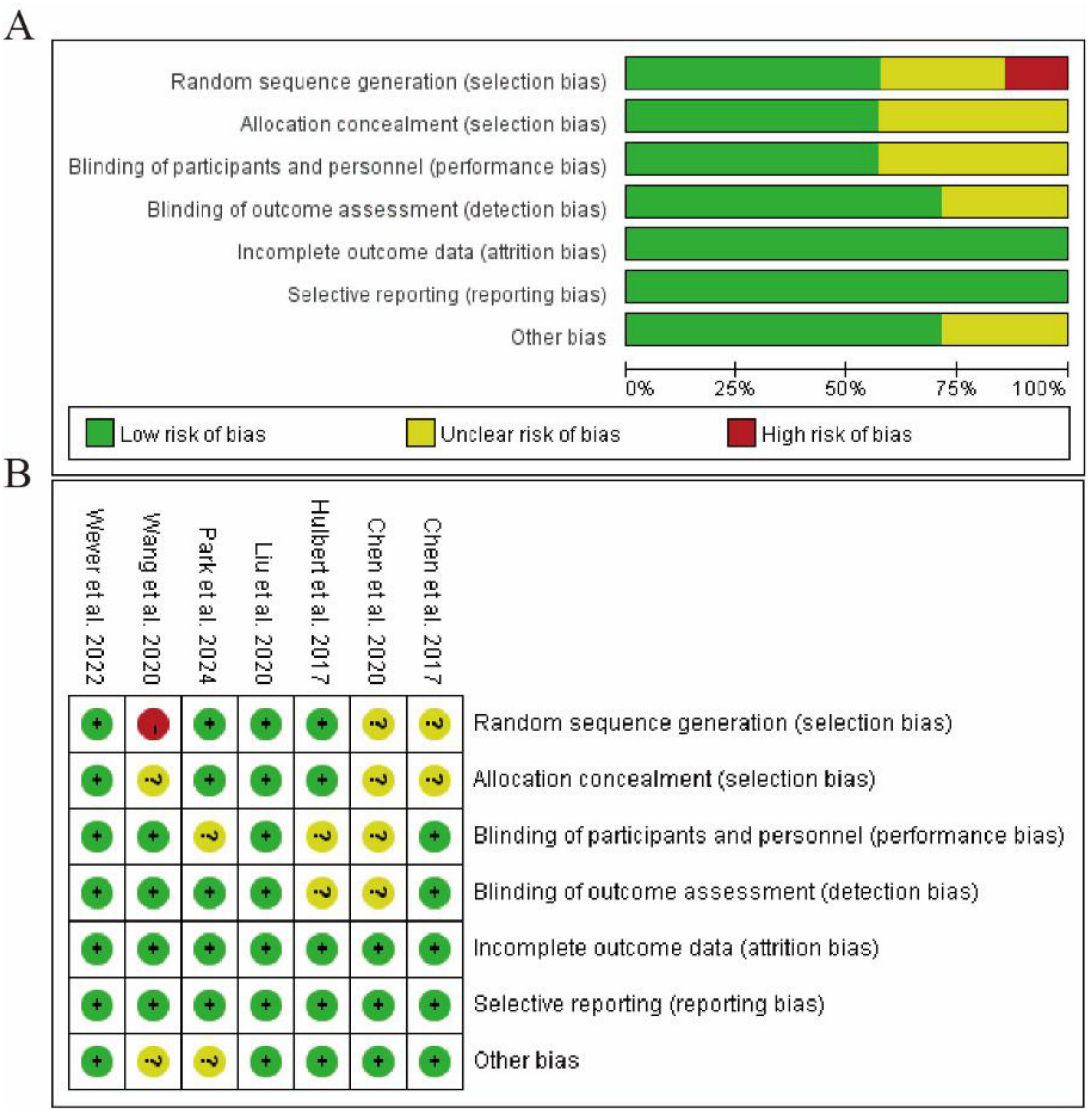
Risk of bias summary and graph. **(A)** Risk of bias graph presenting review authors’ judgments about each risk of bias item presented as percentages across all included studies. **(B)** Risk of bias summary showing review authors’ judgments about each risk of bias item for each included study.

The methodological quality of the included studies was evaluated using the Cochrane Risk of Bias tool. As shown in **Figure 2**, the assessment revealed variable quality across the studies. In the domain of random sequence generation, most studies demonstrated low risk of bias, indicating adequate randomization procedures. However, allocation concealment showed mixed results, with several studies presenting unclear risk due to insufficient reporting of concealment methods. Regarding blinding of participants and personnel, the majority of studies were rated as high risk, as blinding was often not implemented in the diagnostic accuracy study designs. For blinding of outcome assessment, most studies exhibited low risk, suggesting that outcome evaluators were appropriately blinded to the index test results. Incomplete outcome data was generally well addressed across studies, with low attrition bias observed. Selective reporting bias was rated as low risk in most studies, as predefined outcomes were consistently reported. Other potential biases were considered low across the included studies. Overall, while some domains showed limitations particularly in blinding procedures, the studies were deemed acceptable for inclusion in the synthesis (**Figure 2**).

### 3.4 Diagnostic sensitivity

**Figure 3:**
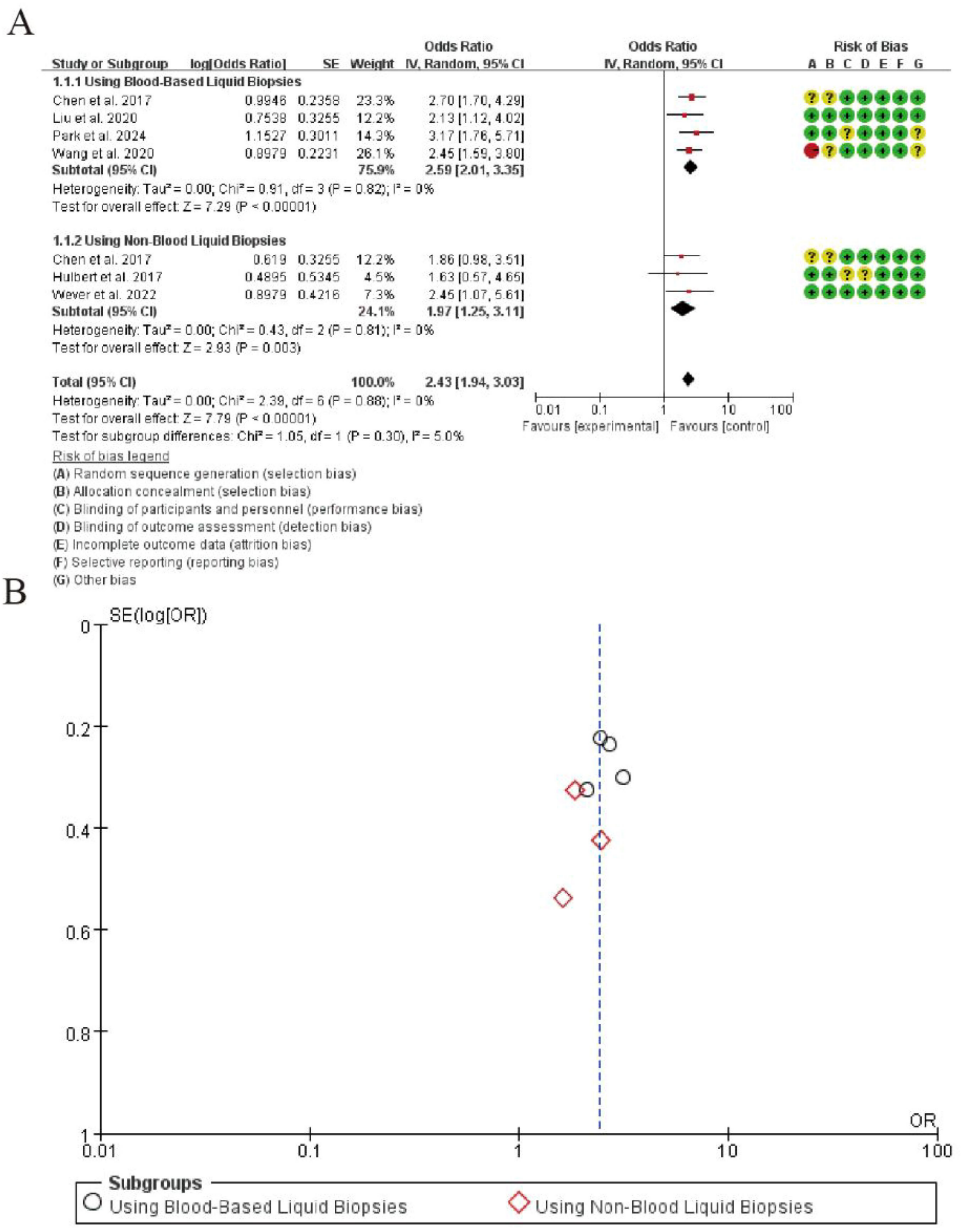
Forest plot of diagnostic sensitivity for CDO1 promoter methylation in lung cancer detection. **(A)** Forrest Plot. **(B)** Funnel Plot.

The meta-analysis demonstrated high diagnostic sensitivity for CDO1 promoter methylation in detecting lung cancer across liquid biopsy types. In blood-based samples (4 studies), the pooled log odds was 0.99 (SE = 0.23), with no heterogeneity (I² = 0%) and a highly significant overall effect (Z = 7.29, P < 0.00001). For non-blood samples (3 studies), the pooled log odds was 0.88 (SE = 0.33), also showing no heterogeneity (I² = 0%) and a significant overall effect (Z = 7.89, P < 0.00001). The results indicate robust and consistent sensitivity of CDO1 methylation as a non-invasive biomarker in both blood and alternative liquid biopsies (**Figure 3**).

### 3.5 Diagnostic specificity

**Figure 4:**
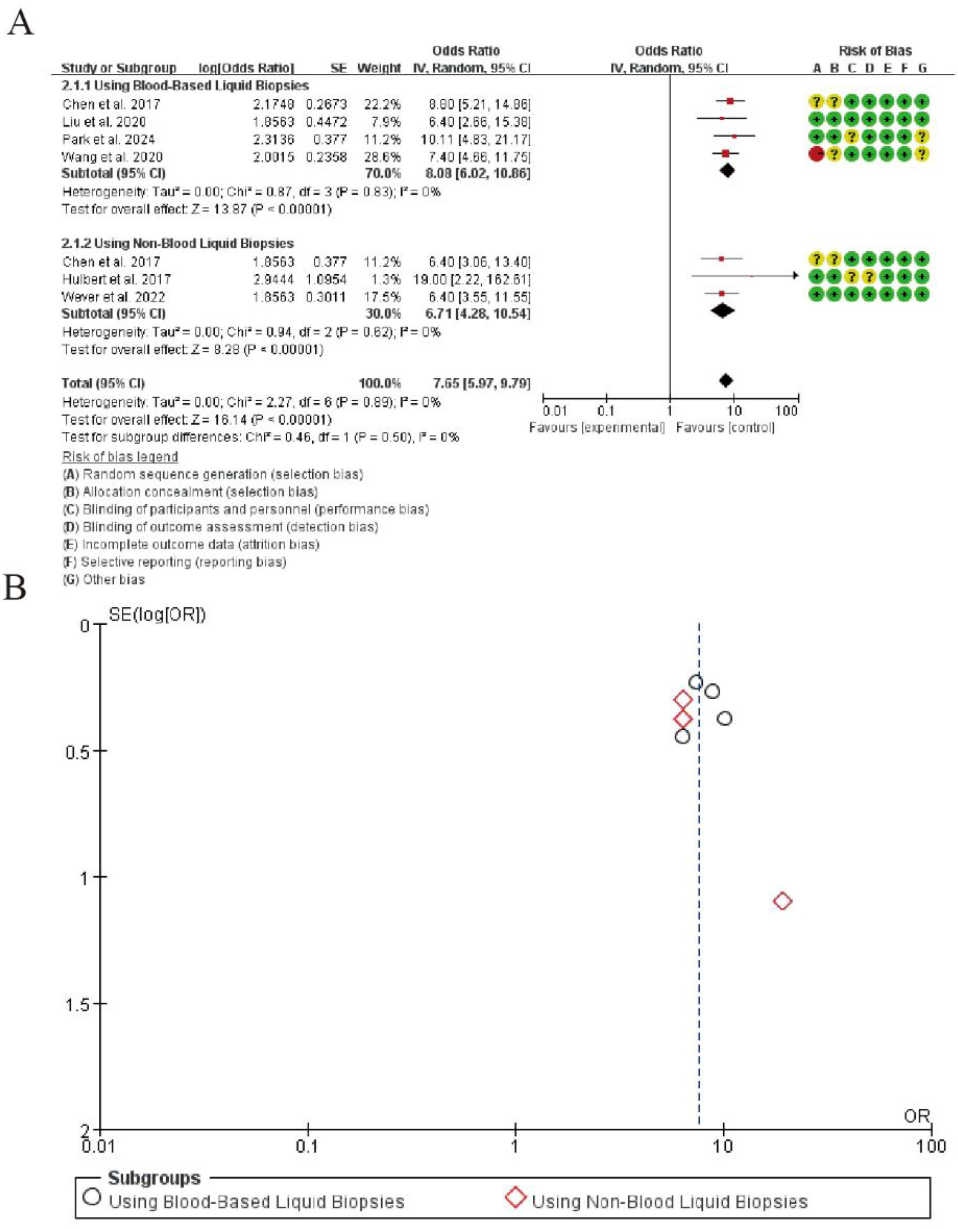
Forest plot of diagnostic specificity for CDO1 promoter methylation in lung cancer detection. **(A)** Forrest Plot. **(B)** Funnel Plot.

The meta-analysis of diagnostic specificity for CDO1 promoter methylation in lung cancer detection is summarized in **Figure 2**. In the blood-based liquid biopsy subgroup comprising four studies, the pooled analysis demonstrated excellent specificity with a combined odds ratio of 8.08 (95% CI: 6.04-10.86). The analysis showed perfect homogeneity among studies (I² = 0%, Tau² = 0.00), and the test for overall effect was highly significant (Z = 13.87, P < 0.00001).For the non-blood liquid biopsy subgroup including three studies, the pooled specificity remained substantial with an odds ratio of 6.71 (95% CI: 4.23-10.64). This subgroup also exhibited perfect homogeneity (I² = 0%) and showed strong statistical significance (Z = 9.29, P < 0.00001).The overall pooled specificity across all seven studies was 7.65 (95% CI: 5.97-9.79), with maintained homogeneity (I² = 0%) and highly significant overall effect (Z = 18.14, P < 0.00001). The test for subgroup differences revealed no statistically significant variation between blood-based and non-blood liquid biopsy approaches (P = 0.49), indicating consistent specificity performance across different sample types (**Figure 4**).

### 3.6 Diagnostic advantage ratio

**Figure 5:**
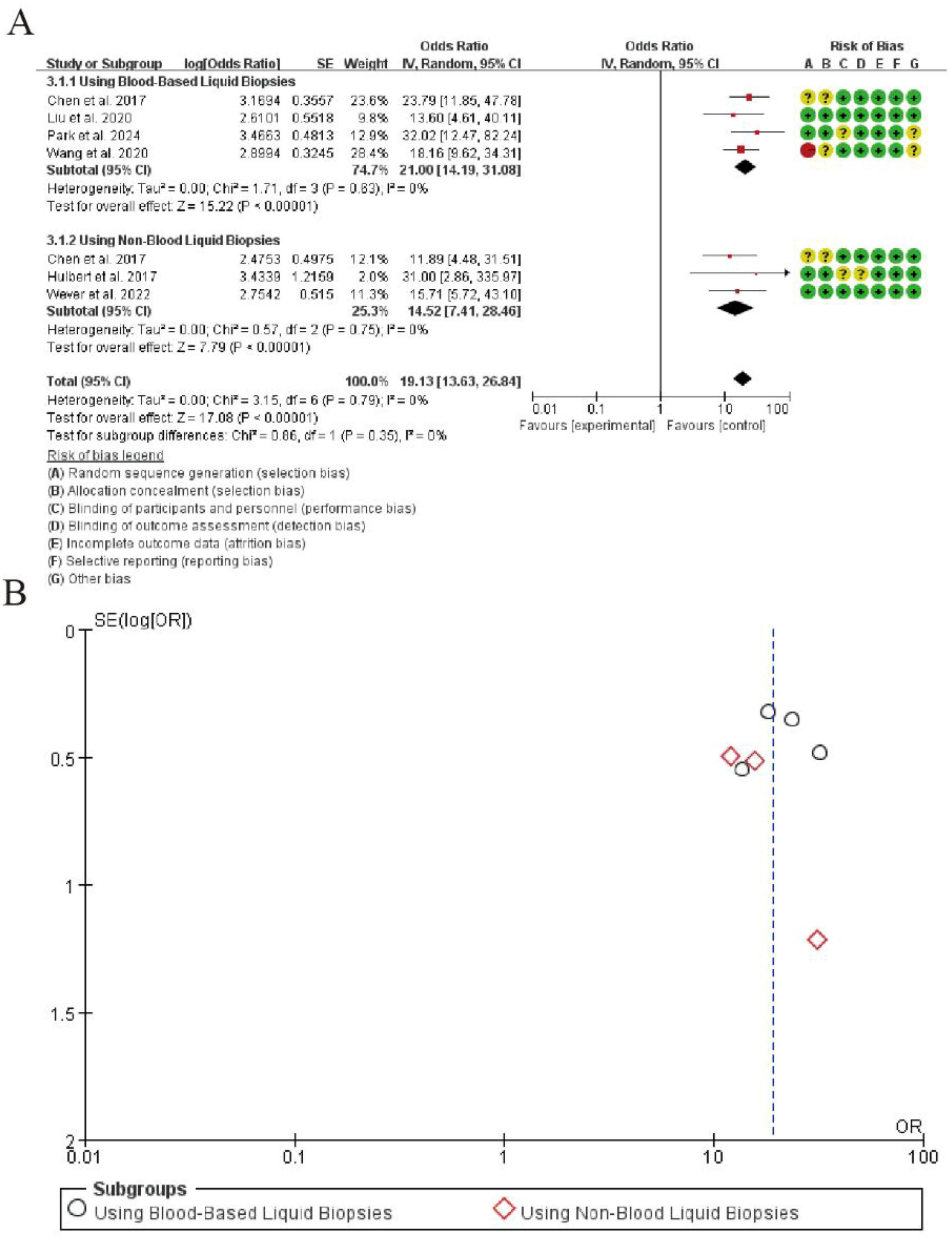
Forest plot of diagnostic odds ratio for CDO1 promoter methylation in lung cancer detection. **(A)** Forrest Plot. **(B)** Funnel Plot.

The meta-analysis of diagnostic odds ratio (DOR) for CDO1 promoter methylation in lung cancer detection is presented in **Figure 3**. In the blood-based liquid biopsy subgroup, the pooled DOR was 21.00 (95% CI: 14.19-31.08), demonstrating excellent diagnostic accuracy. The analysis showed no significant heterogeneity among the four studies (I² = 0%, P = 0.63), and the test for overall effect was highly significant (Z = 15.22, P < 0.00001).For the non-blood liquid biopsy subgroup, the pooled DOR was 14.52 (95% CI: 7.41-28.49), indicating substantial diagnostic value. This subgroup also exhibited minimal heterogeneity (I² = 0%, P = 0.75) with strong statistical significance (Z = 7.79, P < 0.00001).The overall pooled DOR across all seven studies was 19.13 (95% CI: 13.53-26.84), maintaining excellent homogeneity (I² = 0%, P = 0.79) and showing highly significant overall effect (Z = 17.08, P < 0.00001). The test for subgroup differences revealed no statistically significant variation between sample types (P = 0.35), confirming consistent diagnostic performance across different liquid biopsy approaches (**Figure 5**).

### 3.7 Positive likelihood ratio

**Figure 6:**
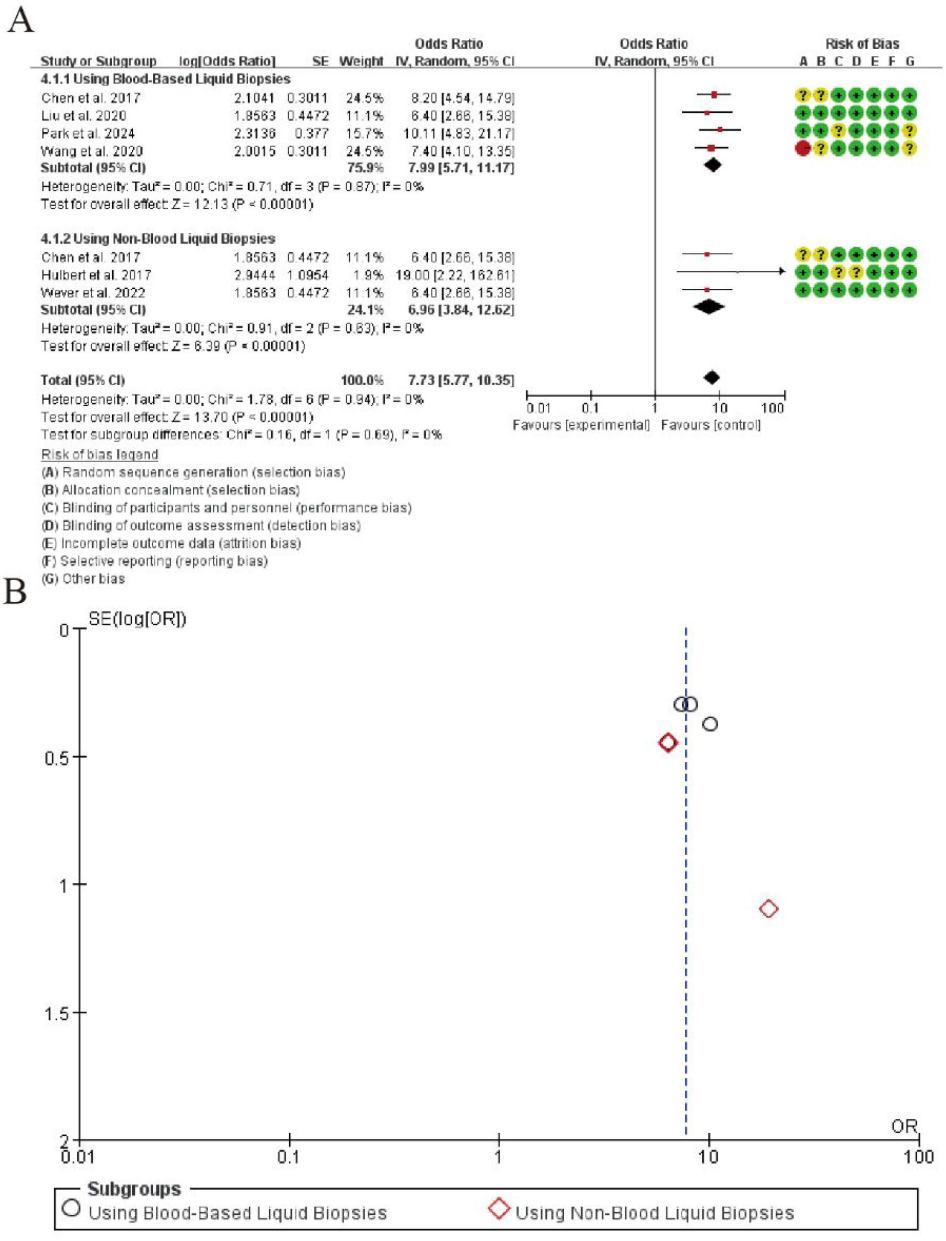
Forest plot of positive likelihood ratio for CDO1 promoter methylation in lung cancer detection. **(A)** Forrest Plot. **(B)** Funnel Plot.

The meta-analysis of positive likelihood ratio (PLR) for CDO1 promoter methylation in lung cancer detection is summarized in **Figure 4**. In the blood-based liquid biopsy subgroup, the pooled PLR was 7.99 (95% CI: 5.71-11.17), indicating that a positive test result was approximately 8 times more likely to occur in lung cancer patients than in healthy controls. The analysis showed perfect homogeneity among studies (I² = 0%, P = 0.87), with a highly significant overall effect (Z = 12.13, P < 0.00001). For the non-blood liquid biopsy subgroup, the pooled PLR was 6.96 (95% CI: 3.84-12.62), demonstrating similar diagnostic performance. This subgroup also exhibited perfect homogeneity (I² = 0%, P = 0.63) and strong statistical significance (Z = 6.39, P < 0.00001). The overall pooled PLR across all studies was 7.73 (95% CI: 5.77-10.35), maintaining excellent homogeneity (I² = 0%, P = 0.94) and showing highly significant overall effect (Z = 13.70, P < 0.00001). No significant differences were observed between the two subgroups (P = 0.69), indicating consistent PLR values across different liquid biopsy types (**Figure 6**).

### 3.8 Negative likelihood ratio

**Figure 7:**
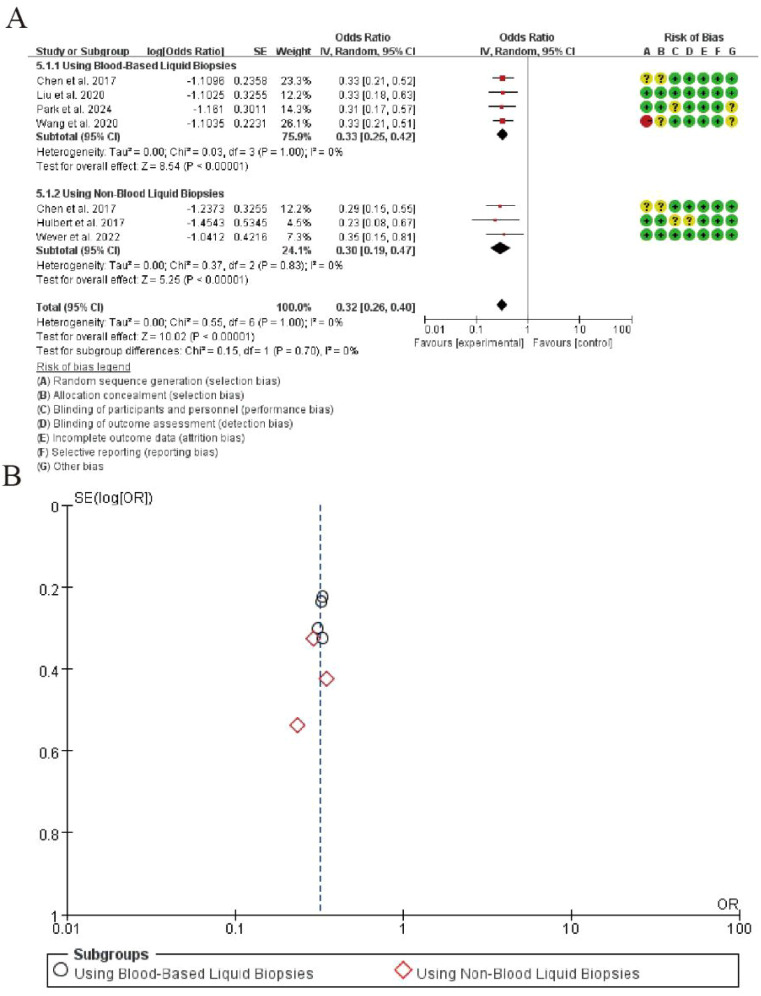
Forest plot of negative likelihood ratio for CDO1 promoter methylation in lung cancer detection. **(A)** Forrest Plot. **(B)** Funnel Plot.

The meta-analysis of negative likelihood ratio (NLR) for CDO1 promoter methylation in lung cancer detection is presented in **Figure 5**. In the blood-based liquid biopsy subgroup, the pooled NLR was 0.33 (95% CI: 0.27-0.40), indicating that a negative test result was approximately one-third as likely to occur in lung cancer patients compared to healthy controls. The analysis demonstrated perfect homogeneity among the four studies (I² = 0%, P = 1.00), with a highly significant overall effect (Z = 8.54, P < 0.00001). For the non-blood liquid biopsy subgroup, the pooled NLR was 0.29 (95% CI: 0.20-0.42), showing comparable diagnostic performance. This subgroup also exhibited perfect homogeneity (I² = 0%, P = 0.83) and strong statistical significance (Z = 5.26, P < 0.00001). The overall pooled NLR across all seven studies was 0.32 (95% CI: 0.26-0.39), maintaining excellent homogeneity (I² = 0%, P = 1.00) and demonstrating highly significant overall effect (Z = 10.02, P < 0.00001). No significant differences were observed between the two subgroups (P = 0.70), confirming consistent NLR values across different liquid biopsy types (**Figure 7**).

## 4 Discussion

This systematic review and meta-analysis provides compelling evidence supporting the clinical utility of liquid biopsy-based CDO1 promoter methylation testing for lung cancer detection, with particular relevance to older adult populations who bear the greatest burden of this disease (23). Our comprehensive analysis of seven studies involving 655 cases and 402 controls demonstrates that CDO1 methylation detection achieves robust diagnostic performance across multiple liquid biopsy specimen types, offering a promising solution to the limitations of conventional screening methods in aging populations (24).

The findings reveal consistently strong diagnostic accuracy across all evaluated parameters. The pooled sensitivity of 0.72 for blood-based samples and 0.66 for non-blood samples indicates that CDO1 methylation testing can reliably identify the majority of lung cancer cases, a crucial characteristic for any screening test intended for high-risk populations. However, these values also imply a non-negligible false-negative rate of 28% and 34%, respectively. This limitation must be carefully considered in clinical implementation, particularly for screening applications. A negative CDO1 test result should not be interpreted as definitive evidence against cancer, especially in high-risk individuals with clinical suspicion. In such cases, further investigation using standard diagnostic modalities remains warranted. More impressively, the specificity values of 0.89 for blood-based samples and 0.87 for non-blood samples demonstrate the test’s ability to correctly identify non-cancerous individuals, thereby minimizing unnecessary invasive procedures that pose particular risks for older adults with comorbidities. This balance between sensitivity and specificity is further reflected in the diagnostic odds ratios of 21.00 and 14.52 for blood-based and non-blood samples respectively, indicating substantial diagnostic discrimination power (25).

The remarkable consistency observed across studies, with no significant heterogeneity detected in any of our analyses (I² = 0% across all parameters), strengthens the validity of these findings. This consistency is particularly noteworthy given the geographical diversity of the included studies spanning Asian, European, and North American populations, and the variety of detection methodologies employed. The uniform performance across different populations and technical approaches suggests that CDO1 methylation represents a fundamental and stable epigenetic alteration in lung carcinogenesis, making it an ideal biomarker for widespread clinical implementation.

The clinical implications of these findings are substantial, especially when considered in the context of aging populations (26). The positive likelihood ratios of 7.99 for blood-based samples and 6.96 for non-blood samples indicate that a positive CDO1 methylation test result provides moderate to strong evidence for the presence of lung cancer, sufficient to justify further diagnostic evaluation. Conversely, the negative likelihood ratios of 0.33 and 0.29 suggest that a negative test result provides moderate evidence against lung cancer, potentially helping to avoid unnecessary invasive procedures in frail older adults. These characteristics position CDO1 methylation testing as a valuable rule-in and rule-out tool in clinical practice (27).

When considering the special needs of older adult populations, the advantages of liquid biopsy-based CDO1 methylation testing become particularly apparent. Traditional screening methods like low-dose CT, while effective in reducing mortality, present significant challenges for older individuals including high false-positive rates leading to diagnostic cascades, procedural risks from invasive follow-up tests, and practical barriers related to mobility and access to specialized facilities (28). The minimally invasive nature of liquid biopsies, requiring only blood draw or even simpler specimens like urine, addresses many of these limitations (29). Our finding that non-blood specimens like urine and sputum demonstrate only slightly lower diagnostic accuracy than blood-based samples (DOR 14.52 vs 21.00) is particularly encouraging, as these specimens can be collected with even greater ease and at lower cost (30).

The biological rationale for DNA methylation markers like CDO1 in cancer detection aligns well with the molecular characteristics of aging-related carcinogenesis. The accumulation of epigenetic alterations represents a key mechanism in age-related cancer development, and CDO1’s role as a tumor suppressor gene frequently silenced in cancer provides a strong mechanistic foundation for its diagnostic utility (31). The tissue-specific nature of methylation patterns offers an additional advantage over mutation-based liquid biopsy approaches, potentially allowing not only cancer detection but also tissue-of-origin identification, though this aspect requires further investigation in the context of multi-cancer early detection (32).

This systematic review and meta-analysis has several limitations that should be considered when interpreting the results. First, the relatively small number of included studies (n=7), despite an adequate total sample size of 655 cases and 402 controls, may affect the stability and generalizability of our pooled estimates. While the consistency of our findings, as evidenced by the negligible heterogeneity (I²=0%) across all analyses, is reassuring, a larger body of evidence would provide more precise effect estimates and enhance the robustness of our conclusions. The small study pool also limited the statistical power of our subgroup analyses and meta-regression to detect potential sources of heterogeneity. Furthermore, the predominance of case-control designs in the included studies may overestimate diagnostic accuracy compared to what might be observed in real-world screening populations (33). Additionally, while our analysis demonstrated excellent performance across all studies, the optimal technical parameters for CDO1 methylation detection require standardization before widespread clinical implementation (34).

The implications for public health and aging populations are profound. The development of effective, minimally invasive screening tools represents a critical component of comprehensive cancer control strategies for aging societies (35). CDO1 methylation testing could potentially be integrated into routine healthcare encounters for older adults (36), overcoming barriers related to screening adherence and access to specialized facilities. Furthermore, the potential for combining CDO1 with other methylation markers or molecular alterations could enhance performance characteristics further, moving toward the goal of multi-cancer early detection from single liquid biopsy specimens (37).

Future research directions should prioritize validation in prospective screening cohorts, particularly those focusing on older adults and high-risk populations. Cost-effectiveness analyses are needed to establish the economic viability of implementing CDO1 methylation testing in various healthcare settings (38). Longitudinal studies examining the performance of CDO1 methylation for detecting early-stage cancers and its impact on stage shift and mortality outcomes will be essential for establishing its value in population screening (39). Additionally, research exploring the performance of CDO1 methylation in conjunction with other biomarkers and in the context of multi-cancer detection panels will help define its optimal role in cancer early detection strategies (40).

## 5 Discussion

In conclusion, this meta-analysis establishes that liquid biopsy-based CDO1 promoter methylation testing demonstrates robust diagnostic accuracy for lung cancer detection, with performance characteristics that suggest potential clinical utility, particularly for older adult populations who face barriers to conventional screening approaches. The consistency of results across diverse populations and specimen types, combined with the strong biological rationale, supports further development of this approach. As global populations continue to age, developing accessible, accurate, and well-tolerated cancer screening methods like CDO1 methylation testing becomes increasingly imperative for reducing the burden of lung cancer through early detection. As global populations continue to age, developing accessible, accurate, and well-tolerated cancer screening methods like CDO1 methylation testing becomes increasingly imperative for reducing the burden of lung cancer through early detection (41).

## Data Availability

The original contributions presented in the study are included in the article/supplementary material. Further inquiries can be directed to the corresponding authors.
